# Subclinical cardiac damage in cancer patients before chemotherapy

**DOI:** 10.1007/s10741-021-10151-4

**Published:** 2021-07-27

**Authors:** Iacopo Fabiani, Giorgia Panichella, Alberto Aimo, Chrysanthos Grigoratos, Giuseppe Vergaro, Nicola Riccardo Pugliese, Stefano Taddei, Daniela Maria Cardinale, Claudio Passino, Michele Emdin, Alberto Giannoni

**Affiliations:** 1 Cardiology Division, Fondazione Toscana Gabriele Monasterio, Pisa, Italy; 2 Institute of Life Sciences, Scuola Superiore Sant’Anna, Pisa, Italy; 3 Internal Medicine Unit, University Hospital of Pisa, Pisa, Italy; 4 Cardioncology Unit, Cardiology Division, European Institute of Oncology, I.R.C.C.S, Milan, Italy

**Keywords:** Cancer, Cardiovascular disease, Heart failure, Cardiac toxicity

## Abstract

Cancer and cardiovascular diseases, including heart failure (HF), are the main causes of death in Western countries. Several anticancer drugs and radiotherapy have adverse effects on the cardiovascular system, promoting left ventricular dysfunction and ultimately HF. Nonetheless, the relationship between cancer and HF is likely not unidirectional. Indeed, cancer and HF share common risk factors, and both have a bidirectional relationship with systemic inflammation, metabolic disturbances, and neurohormonal and immune activation. Few studies have assessed the impact of untreated cancer on the heart. The presence of an active cancer has been associated with elevated cardiac biomarkers, an initial impairment of left ventricular structure and function, autonomic dysfunction, and reduced exercise tolerance. In turn, these conditions might increase the risk of cardiac damage from chemotherapy and radiotherapy. HF drugs such as beta-blockers or inhibitors of the renin–angiotensin–aldosterone system might exert a protective effect on the heart even before the start of cancer therapies. In this review, we recapitulate the evidence of cardiac involvement in cancer patients naïve from chemotherapy and radiotherapy and no history of cardiac disease. We also focus on the perspectives for an early diagnosis and treatment to prevent the progression to cardiac dysfunction and clinical HF, and the potential benefits of cardioactive drugs on cancer progression.

## Introduction

Recent advances in cancer therapies have considerably improved the survival rates of many cancers [1], but several anticancer drugs and radiotherapy regimens have detrimental effects on the heart, leading to pericardial and myocardial disease, left ventricular (LV) dysfunction, and ultimately heart failure (HF) [2]. In this perspective, there is a growing interest in the bidirectional relationship between cancer and HF, beyond the possible effects of cancer therapies. Cancer per se might promote HF development, whereas HF could act as a pro-oncogenic condition [3]. Cancer and HF share several risk factors, such as hypertension, diabetes, obesity, and smoking [4–6], and the systemic metabolic alterations could link one disease to the other [7, 8]. While there is evidence that patients with HF have a higher incidence of cancer compared to the general population [9], data on the association between cancer alone (i.e., naïve to therapies) and HF are very limited. Until now, research mainly focused on the long-term risk of cardiovascular disease in cancer survivors [10–12].

In this review, we discuss the main evidence of subclinical cardiac damage in treatment-naïve cancer patients, and recapitulate the possible mechanisms leading to HF, including inflammation, oxidative stress, and autonomic impairment. We also discuss the potential roles of drug therapies for the prevention of cancer-related cardiotoxicity, and the potential benefits of cardioactive drugs on cancer progression.

## Possible mechanisms leading to HF in cancer patients

**Fig. 1:**
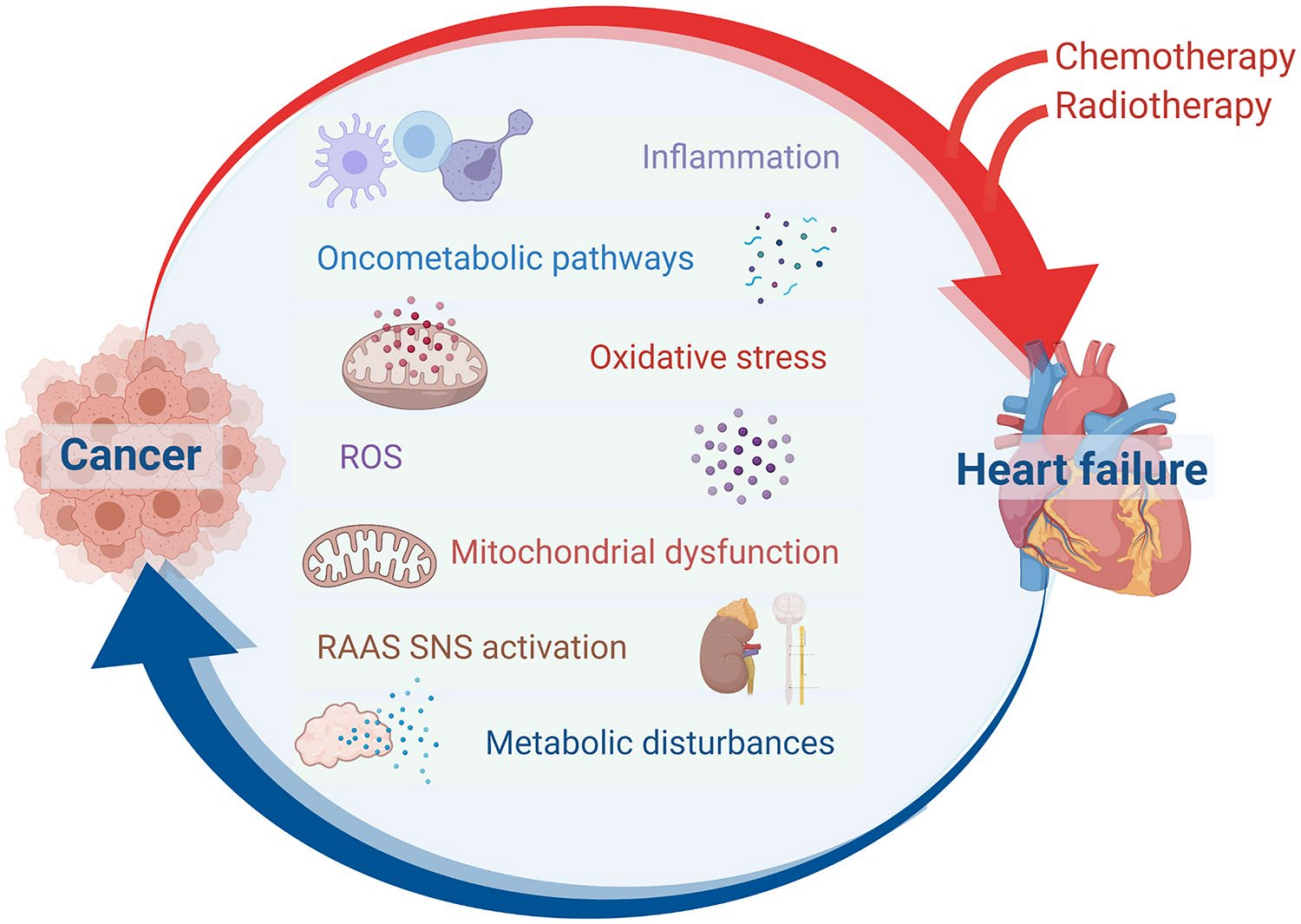
Pathophysiological interplay heart-cancer disease in patients naïve to chemotherapy. Cancer and heart have a bidirectional relationship (cancer to heart; heart to cancer). Besides a direct effect mediated by chemo (and radio) therapy, cancer can impact on heart structure and function in several different pathways: inflammation, oxidative stress, metabolic (oncometabolites), and directly affecting sympathetic nervous system (SNS) activation

Systemic inflammation and oxidative stress, neurohormonal activation, autonomic dysfunction, clonal haematopoiesis, and metabolic derangements are the main proposed mechanisms whereby cancer may promote cardiac dysfunction, and, ultimately, HF (Fig. 1).

Several conditions have detrimental effects on the heart and may lead to HF. Systemic amyloidoses are a group of diseases characterized by the deposition of amyloid, an aggregate of misfolded proteins, in one or more organs. The two most common forms of cardiac amyloidosis are due transthyretin amyloidosis (ATTR) and light-chain amyloidosis (AL), derived from abnormal light chains produced by plasma cell malignancies [13]. Pheochromocytomas, rare catecholamine-secreting tumours of the adrenal glands, often cause a severe cardiotoxicity mediated by β adrenergic receptor stimulation, which manifests in several forms, from Takotsubo to dilated cardiomyopathy [14, 15]. Carcinoid heart disease is the cardiac involvement of serotonin-producing neuroendocrine tumours (NETs), due to fibrotic endocardial plaques with associated valve dysfunction often leading to right-sided HF [16]. In acromegaly, the excess of growth hormone (GH) and insulin-like growth factor 1 (IGF-I) induces a specific cardiomyopathy whose most common feature is concentric hypertrophy, usually associated with diastolic dysfunction and eventual impairment of systolic function and HF [17].

### Inflammation and oxidative stress

Most malignancies elicit an inflammatory response through the release of proinflammatory cytokines and acute phase proteins that create a protumorigenic microenvironment, which in turn contributes to cancer invasiveness [18–23]. Inflammation also promotes microvascular endothelial dysfunction and HF development (particularly HF with preserved ejection fraction) in experimental models, and proinflammatory cytokines (such as tumour necrosis factor-alpha [TNFα], interleukin-1β and -6) may reduce contractility and promote adverse LV remodelling [3, 24, 25].

Oxidative stress, due for example to reactive oxygen species (ROS), works synergistically with inflammation to promote both cancer and HF [26]. Sustained exposure to ROS can cause DNA damage and further support cancer promotion and progression [27–29]. Cancer itself can foster oxidative stress, mainly through the effect of oncogenes (e.g., Myc or Ras) affecting cellular metabolism, redox homeostasis, and DNA replication [30]. Similarly, mitochondrial dysfunction is a prominent feature of HF, resulting in increased cellular levels of ROS and reactive nitrogen species (RNS), altered calcium handling, mitochondrial DNA replication, excitation–contraction coupling, promotion of cardiomyocyte hypertrophy and apoptosis, and myocardial fibrosis [31, 32].

### Renin–angiotensin–aldosterone system activation

Increased rennin-angiotensin-aldosterone system (RAAS) activity has been demonstrated in various tumour types, including kidney, prostate, bladder, stomach, cervix, brain, pancreas, colon, lung, liver, skin, and haematopoietic cancers [33, 34]. Angiotensin receptor-1 (ATR1) signalling appears to be the major component of RAAS involved in tumour growth (by inducing angiogenesis) and tumour proliferation (by promoting vascular or epidermal growth factor receptor expression) [33, 35]. ATR1 stimulates inflammation, fibrosis, angiogenesis, tumour invasion and metastasis, while ATR2 antagonize these effects [33, 35]. Angiotensin II can also promote cell growth and proliferation through the transforming growth factor-beta [36], tyrosine kinase [37], and activating mammalian target of rapamycin (mTOR) pathways [38]. Furthermore, a subpopulation of cancer cells known as cancer stem cells (CSCs) has been identified in many types of cancers [39]. These cells express components of the RAAS, supporting the intriguing hypothesis of paracrine mechanisms fostering carcinogenesis via stem cells [40].

The RAAS has a well-established pathogenetic role in HF [41, 42], promoting myocardial hypertrophy and fibrosis and adverse LV remodelling. Thus, enhanced RAAS activity in cancer patients might promote HF development.

### Cardiac autonomic dysfunction

The possible occurrence of autonomic dysfunction in cancer is increasingly acknowledged. It is particularly prevalent among patients with advanced cancer and in those undergoing chemotherapy and radiotherapy, but studies suggest an increased prevalence among cancer patients compared to cancer-free individuals. Autonomic dysfunction could be related to preexisting neuropathy, paraneoplastic effects, tumour invasion or compression of autonomic nerves, cancer-related deconditioning, or autoimmune disorders [43–45]. Sustained activation of the sympathetic nervous system (SNS) may contribute to cancer initiation [46] and progression [47]. It also influences the tumour microenvironment by promoting the secretion of proinflammatory cytokines [48, 49] and suppressing the immune response [50, 51]. SNS stimulation can also induce cancer cells to escape anoikis, a form of cell death occurring when cells detach from the extracellular matrix [52, 53]. High levels of tissue catecholamines can further promote tumour invasiveness by upregulating matrix metalloproteinases (MMPs) [54, 55], thus increasing vascularisation and matrix degradation, which are the first steps toward metastatic dissemination [24, 56]. Finally, adrenergic stimulation recruits activated macrophages to the tumour parenchyma and induces a prometastatic gene expression signature [57]. These effects are largely mediated by beta-adrenergic receptors, particularly beta-2 adrenoreceptors, and may then be counteracted by beta-blockers [58]. Several preclinical studies show that adrenergic activation modulates apoptosis, promotes angiogenesis and other cancer hallmarks, which provide a rationale for the use of beta-blockers as antineoplastic and cardioprotective agents, and even as adjuvants to cancer chemotherapy [59, 60].

As in the case of RAAS activation, autonomic dysfunction due to cancer might promote the progression to HF. Increased sympathetic outflow and vagal withdrawal is a hallmark of HF [61], particularly HF with reduced ejection fraction (HFrEF), but also mid/range and HFpEF [62]. These changes are initially compensatory, but lead over time to maladaptive ventricular remodelling, hypertrophy, myocardial fibrosis, myocyte cell death, and further deterioration of cardiac function [63].

### Clonal haematopoiesis

Clonal haematopoiesis (CH) is a proliferation of haematopoietic stem cells carrying somatic mutations [64]. These mutations occur predominantly in genes encoding for key epigenetic regulators of haematopoiesis [65, 66]. Some of these somatic mutations in the haematopoietic stem cells are linked to an increased risk of coronary artery disease [66, 67]. Other somatic mutations are associated with a poor prognosis in HF patients [68], with their modulation preventing the worsening of cardiac dysfunction in preclinical mouse models [69]. Accordingly, patients with CH have a higher risk of cancer, HF, and death than controls without mutations [70].

### Oncometabolic pathways

Oncometabolites are molecules that accumulate in cancer cells, often through mutations of genes encoding the corresponding enzymes, which drive the activation of oncogenic pathways [71]. A preclinical study in rodents showed that an increased production of the oncometabolite D-2-hydroxyglutarate by mutant leukemic cells may reduce cardiac contractility by impairing oxidative decarboxylation of α-ketoglutarate and increasing ATP citrate lyase activity [8]. Similarly, results from genetically engineered and nude mice carrying tumours expressing mutant isocitrate dehydrogenase-2 (IDH2) suggest that D-2-hydroxyglutarate promote the development of cardiomyopathy [72].

An energy shift from mitochondrial oxidative phosphorylation to aerobic glycolysis is often observed in cancer cells. This so-called Warburg effect has been extensively studied in cancer, but there is growing evidence of its involvement even in non-tumour diseases, including HF [73, 74]. A shift from the adult to the foetal isoforms of pyruvate kinase (PKM1 to PKM2) is a hallmark of the Warburg effect, with the latter causing accumulation of intermediates of the glycolytic pathway [75]. A high PKM2 expression has been found not only in cancer cells, but also in the failing hearts of patients with advanced HF, and is partially reversible through mechanical unloading [76].

## Evidence of cardiac disease in cancer patients

Characterizing the effects of cancer on the heart requires the assessment of circulating biomarkers, imaging techniques, evaluation of functional capacity and autonomic function (Table 1).

**Table 1:** Subclinical markers of cardiac impairment in cancer patients

Parameter type	Parameter	Patients (number)	Patient characteristics (age, sex, CV risk factors)	Cancer	Main findings	Citation number
Biomarkers	NT-proBNP	145 cancer patients not receiving treatment	Mean age 62.8 years 62% men, 38% women Median LVEF 52.5% Median NT-proBNP 7540 ng/L	Haematologic and solid organ malignancies	In 80% of patients with NT-proBNP > 3000 ng/L, there was evidence of fluid overload	[87]
	hs-cTnT	3.512 cancer survivors	Mean age 76 years 38% men, 62% women Diabetes 30%, Hypertension 70%	Different cancers, especially breast, prostate and colorectal cancer	Cancer survivors had significantly higher odds of elevated hs-cTnT	[89]
	NT-proBNP, MR-proANP, MR-proADM, CT-proET-1, copeptin, hsTnT, IL-6, CRP, SAA, haptoglobin, fibronectin	555 treatment-naïve cancer patients	Mean age 62 years 40% men, 60% women Hypertension 45%, diabetes 8%, CVD 12%	Many types of cancer	All CV hormones and hs-TnT levels rose with tumour stage progression. All markers were independent predictors of mortality	[92]
	hs-TnT	30 patients before starting ICI therapy	Median age 68 years 77% men, 23% women CAD 13%, Current/former smokers 90%, Overweight/obese 67%, Hypertension 43%	Lung cancer	Only patients with baseline hs-TnT ≥ 14 ng/L died, had a stroke/TIA, or new-onset HF. 9/13 patients with progression of cardiac disease had baseline hs-TnT ≥ 14 ng/L	[88]
Exercise capacity	VO_2peak_	248	All women Median age 55 ± 8 years	Breast cancer	Breast cancer patients have marked impairment in VO_2peak_, which may be an independent predictor of survival in metastatic disease	[111]
Automatic function	RHR	548 treatment-naïve cancer patients	Median age 62 years 40.9% men, 32.7% women Hypertension 45%, diabetes 8%, CVD 5%	Breast, lung and gastrointestinal cancer, myelodysplastic and myeloproliferative diseases	↑RHR display ↑ CV biomarkers RHR was associated with all-cause mortality, especially in lung and gastrointestinal cancers	[115]
	HRV	383 gastric cancer patients	Median age 60.72 ± 11.82 years 71.5% men, 28.5% women	Gastric cancer	↓HRV correlates with tumour stage and progression	[120]
	VO_2peak_, LVEF, lean mass, HRV	50 patients with CRC 51 with HF 51 controls	Median age 59.9 ± 12.0 40% men, 60% women Hypertension, diabetes	Colorectal cancer	↓ LVEF and ↓VO_2peak_ in CRC patients. Exercise capacity, LVEF, lean mass, and HRV were impaired in chemotherapy-treated and -naive patients	[91]

*CAD* coronary artery disease, *CRC* colorectal cancer, *CRP* C-reactive protein, *CT-proET-1* C-terminal proendothelin-1, *CV* cardiovascular, *HF* heart failure, *HRV* heart rate variability, *hs-cTnT* high-sensitivity cardiac troponin T, *ICI* immune checkpoint inhibitor, *IL-6* interleukin 6, *LVEF* left ventricular ejection fraction, *MR-proADM* mid-regional proadrenomedullin, *MR-proANP* mid-regional proatrial natriuretic peptide, *NT-proBNP* N-terminal pro-B-type natriuretic peptide, *RHR* resting heart rate, *SAA* serum amyloid A, *VO*_*2peak*_ peak oxygen cardiac consumption

### Biomarkers

Laboratory markers can be used to assess a variety of pathophysiological processes in HF, such as fibrosis, inflammation, myocardial injury, and remodelling [77]. There is extensive literature on the role of cardiac biomarkers (particularly natriuretic peptides and high-sensitivity troponins [hs-Tn]) for the early detection of cardiotoxicity from cancer therapies [78–80]. Much less is known about circulating levels and prognostic value of biomarkers before the start of chemotherapy.

Recent studies reported elevated levels of several biomarkers of myocardial injury and alterations of immunity and inflammatory pathways in treatment-naïve cancer patients [81–88]. A retrospective study in 145 patients with haematologic and solid organ malignancies not on treatment investigated the levels of *N*-terminal pro-B-type natriuretic peptide (NT-proBNP). There was evidence of fluid overload in 80% of patients with elevated NT-proBNP. The degree of NT-proBNP elevation was similar between patients with and without HF or volume overload and those with solid vs haematologic malignancies [87]. The role of high-sensitivity troponin (hs-Tn) levels to detect subclinical myocardial damage beyond conventional cardiovascular risk factors has been confirmed in a recent study, including 3512 individuals free from cardiovascular disease [89]. Cancer survivors (19% of the whole population) had significantly higher odds of elevated hs-TnT than patients without prior cancer (odds ratio [OR] 1.26; 95% confidence interval [CI], 1.03–1.53). Furthermore, a significant increase of hs-TnI in 25 haematologic patients naïve from anthracycline therapy has been reported [90]. Overall, despite an association between increased NT-proBNP or hs-Tn, there is limited evidence of a link between this increase and alterations of cardiac structure or function in treatment-naïve cancer patients [91].

A prognostic role of biomarkers in this setting has also been proposed. In a study on 555 patients with a primary diagnosis of cancer and no prior therapies, several biomarkers (including NT-proBNP, hs-TnT, mid-regional proatrial natriuretic peptide, proadrenomedullin, copeptin, and interleukin 6) were shown to increase with tumour progression. All biomarkers predicted mortality regardless of age, gender, tumour entity and stage, and cardiac comorbidities [92]. Recently, hs-TnT before the start of treatment was reported to predict a composite endpoint of cardiovascular death, stroke or transient ischemic attack, pulmonary embolism and new-onset HF, as well as the progression of cardiac involvement at 3 months, in patients on immune checkpoint inhibitors, with 14 ng/L as the best cutoff [88].

In summary, patients with cancer can display an increase in cardiac biomarkers before the start of cancer therapies, and such increase might predict an increased risk of adverse outcome. However, further studies are needed to assess the prognostic role of other cardiac biomarkers, such as soluble suppression of tumorigenicity-2, galectin-3, or fibroblast growth factor-23.

### Imaging findings

Echocardiography is the first-line imaging technique to assess cardiac structure and function. Although LV ejection fraction (LVEF) is the most commonly used parameter to detect cardiotoxic damage [79], speckle tracking imaging has emerged as a more sensitive marker of subclinical myocardial dysfunction [79, 93–95], with prognostic relevance in terms of prediction of manifest LV dysfunction [96–99].

Cramer et al. reported increased end-systolic volumes but similar diastolic function parameters in patients with evidence of colorectal cancer (CRC) before chemotherapy [91] compared with controls, but their analysis did not include advanced echocardiographic imaging. A retrospective study on 122 chemotherapy and radiotherapy-naive patients with cancer and 45 controls with similar cardiovascular risk profile showed that cancer, even before the initiation of therapy, was associated with reduced longitudinal (OR 9.0; 95% CI, 2.20–23.50; *p* < 0.001), circumferential (OR 7.1; 95% CI, 3.80–20.40; *p* < 0.001), and radial strain (OR 7.2; 95% CI, 3.41–25.10; *p* < 0.001) regardless of age, sex, body mass index, diabetes, and hypertension [100]. Similarly, cancer was independently associated with reduced right ventricle (RV) global longitudinal strain (OR 3.79; 95% CI, 2.18–10.92; *p* < 0.001), as well as with decreased free wall RV longitudinal strain (OR 5.73; 95% CI, 3.17–9.85; *p* < 0.001) [101].

Cardiac magnetic resonance (CMR) allows a characterisation of myocardial tissue changes such as intracellular and interstitial oedema and fibrosis, which may represent early markers of myocardial injury [102]. T1- and T2-weighted imaging and T2 and T1 mapping sequences can help identify intracellular and interstitial oedema [103, 104]. CMR is then a valuable tool for early detection of cardiotoxicity by CMR [105–107]. There is currently no evidence of its prognostic value in chemotherapy and radiotherapy-naïve patients.

### Exercise capacity

Several studies have reported a significant reduction of functional capacity in patients on chemotherapy or cancer survivors [108–110]. The functional implications of cardiorespiratory performance in untreated cancer patients, assessed through cardiopulmonary exercise testing, are less clear. A pilot study evaluated 248 patients with breast cancer before, during, and after adjuvant therapy for the non-metastatic disease, or during therapy for metastatic disease. Patients showed a marked reduction in peak oxygen consumption (VO_2_), especially those with metastatic disease, but also those before therapy, compared to patients after adjuvant therapy and age-matched sedentary healthy women. Peak VO_2_ was an independent predictor of survival, with an adjusted hazard ratio (HR) for death of 0.59 for a VO_2_ peak ≥ 15.4 mL/kg/min (95% CI, 0.29–1.19; *p* = 0.14) [111].

Overall, there is limited evidence of cardiac functional impairment of untreated cancer patients, and findings are basically limited to breast cancer patients.

### Autonomic function

Resting heart rate has a strong association with cancer mortality [112–114]. In a study on 548 treatment-naïve cancer patients, higher resting heart rate was associated with higher NT-proBNP and hs-TnT, and predicted all-cause mortality over a median of 25 months after adjustment for age, gender, tumour type and stage, cardiac status, and haemoglobin (HR for each 5 beats per minute increase 1.10; 95% CI, 1.04–1.16; *p* < 0.001). The strongest associations with mortality were observed in lung and gastrointestinal cancer (*p* = 0.007 and *p* < 0.001, respectively) [115].

Assessment of heart rate variability (HRV), evaluated through ECG Holter monitoring, allows a more comprehensive evaluation of autonomic function. Reduced HRV denotes autonomic dysfunction with sympathetic activation and vagal withdrawal and portends a worse prognosis in several conditions, from cardiovascular to neurodegenerative diseases [116–118]. HRV is reduced in several cancer types [119], and decreased with advanced clinical stage and tumour progression (both *p* < 0.001) in patients with gastric cancer [120]. Cramer et al. prospectively studied 50 patients with CRC, 51 patients with HF, and 51 control subjects. Most metrics of HRV were significantly reduced in CRC patients and HF patients compared with control subjects (all *p* < 0.05).

In summary, cancer patients have a higher heart rate and a reduced HRV, with clinical and prognostic implications.

## Therapeutic perspectives

Several therapies with antifibrotic and antiremodelling effect (mainly RAAS antagonists), autonomic modulation (beta-blockers), and antiinflammatory or antioxidant actions [78, 80] have shown to prevent or relieve chemotherapy cardiotoxicity [121]. Exercise training seems also protective [110, 122, 123], for example reducing NT-proBNP increase and systolic dysfunction in breast cancer patient receiving doxorubicin [124] (Table 2).

**Table 2:** Cardioactive drugs and their effects on tumour progression

Drug tested	Study	Patients (number, age, sex, CV risk factors)	Cancer	Main findings	Citation
BB ACEi	Retrospective cohort study	1779 women 60% > 55 years Obesity 24% Hypertension 32% Diabetes 8%	Early-stage breast cancer	BB exposure—↓ hazard of recurrence and cause-specific mortality. BB combined with ACEi—↓ HR for recurrence and cause-specific mortality than ACEi alone	[139]
BB ACEi ARBs	Retrospective cohort study	18,733 women Median age drug users, 62–63 years, non-users, 56–57 years	Non-metastatic breast cancer	Two BB were associated with increased recurrence rates. ACEi were associated with a slightly increased recurrence hazard. ARBs were not associated with recurrence	[140]
BB	Meta-analysis	20,898 subjects	Breast, prostate, melanoma, ovarian cancer	BB use can be associated with prolonged survival in cancer patients, especially patients with early-stage cancer primarily treated with surgery	[131]
BB	Proof of concept study	466 female patients Median age: hypertensive subgroup 57 years; non-hypertensive subgroup 54.5 years	Breast cancer	BB treated patients showed a significant reduction in metastasis development, tumour recurrence, longer disease-free interval and reduction in breast cancer mortality after 10 years	[125]
ARBs	Meta-analysis	New cancer data (61,590 patients 5 trials) Data on solid organ cancers (68,402 patients 5 trials) Data on cancer deaths (93,515 patients 8 trials)	Solid cancer	ARBs had a significantly increased risk of new cancer occurrence. No statistically significant difference in cancer deaths	[136]
ACEi/ARBs	Meta-analysis	324,168 hypertensive patients from 70 randomised controlled trials	Any cancer	No difference in the risk of cancer with ARBs and ACEi. There was an increased risk with the combination of ACEi plus ARBs	[137]
ACEi/ARBs	Meta-analysis	Data for cancer occurrence (59,004 patients, 10 trials) Data for cancer death (37,515 patients, 7 trials) Data for GI cancer (23,291 patients, 5 trials)	Any cancer	No effect on occurrence of cancer No effect on cancer death	[138]

*ACEi* Angiotensin-converting-enzyme inhibitors, *ARBs* angiotensin-receptor blockers, *BB* beta-blockers, *CV* cardiovascular

### Beta-blockers

Patients on beta-blockers before cancer diagnosis tend to show a reduced disease progression and mortality [125–128]. Indeed, beta-blockers limit (mainly via beta-2-adrenergic receptors) inflammation and metastasis formation [59, 129]. Beta-blockers may then represent an adjuvant therapy strategy with a pleiotropic impact on the primary tumour, its microenvironment, and metastasis formation [128–130].

Powe et al. hypothesised a better outcome for breast cancer patients receiving a beta-blocker for hypertension. They evaluated 3 patient subgroups of 466 consecutive female patients (43 treated with beta-blockers, 49 with other antihypertensives, and 374 non-hypertensive control subgroup) with resectable breast cancer and follow-up of > 10 years. The endpoints were breast cancer survival, disease-free survival, formation of distant metastases, and local tumour recurrence. Patients on beta-blockers showed a significant reduction in metastasis development (*p* = 0.026), tumour recurrence (*p* = 0.001), and longer disease-free survival (*p* = 0.01). Furthermore, the risk of metastasis formation and breast cancer mortality at 10 years were reduced by 57% and 71%, respectively [125].

A recent meta-analysis of 12 studies and more than 20,000 patients reported that beta-blocker therapy is associated with improved overall survival (HR 0.79; 95% CI, 0.67–0.93; *p* = 0.004) and disease-free survival (HR 0.69; 95% CI, 0.53–0.91; *p* = 0.009). The effect size was greater, albeit not significantly different, in patients with low-stage cancer or cancer treated primarily with surgery [131].

### RAAS inhibitors

Angiotensin-converting enzyme inhibitor (ACEi) therapy was originally found to be independently associated with a decreased risk for cancer occurrence in a population-based study including hypertensive patients (HR 0.66; 95% CI, 0.63–0.68; *p* < 0.001) [132]. Studies on larger populations reported a lower cumulative incidence of cancer for angiotensin receptor blocker (ARB) users (HR 0.58, 95% CI 0.55–0.62; *p* 0.001) [133, 134]. RAAS blockade has also been associated with better survival in patients with metastatic renal cell carcinoma [135]. Despite this, several meta-analyses have yielded conflicting results regarding the association between ARB therapy and the risk of new diagnosis of cancer [136–138].

A few studies investigated the effect of beta blockers and RAAS inhibitors. In the LACE Study cohort [women with early-stage breast cancer from the Kaiser Permanente Northern California Cancer Registry], including 1779 women (with 292 cancer recurrences, 174 cancer deaths, and 323 total deaths), 23% of patients were treated with either a beta-blocker and/or ACEi. These drugs were associated with older age, postmenopausal status, tamoxifen therapy, higher body mass index, hypertension, and diabetes. ACEi therapy was surprisingly associated with breast cancer recurrence (HR 1.56; 95% CI, 1.02–2.39; *p* = 0.04), but not cause-specific mortality or overall mortality. On the contrary, beta-blocker therapy was associated with a lower hazard of recurrence and cause-specific mortality (HR 0.86; 95% CI, 0.57–1.32; *p* = 0.49). However, there was no evidence of dose response with either medication. For recurrence and cause-specific mortality, therapy with a beta-blocker and an ACEi was associated with a lower HR for the outcome (HR 1.14 and 1.04, for the respective outcome) than when ACEi alone was used (HR 1.56 and 1.27, respectively) [139].

A further retrospective study including a large population of 18,733 women diagnosed with non-metastatic breast cancer between 1996 and 2003 showed that users of any beta-blocker had a lower recurrence hazard in unadjusted models (HR 0.91; 95% CI, 0.81–1.0) and a slightly higher recurrence hazard in adjusted models (adjusted HR 1.3; 95% CI, 1.1–1.5), with similar associations for exposures defined by receptor selectivity and solubility. Metoprolol and sotalol were associated with increased recurrence rates (adjusted HR: 1.5 metoprolol, 2.0 sotalol). ACEi were associated with a slightly increased recurrence hazard, whereas angiotensin II receptor blockers (ARBs) were not associated with recurrence. The authors concluded that the study did not support the hypothesis that beta-blockers reduced the risk of breast cancer recurrence [140].

Retrospective cohort studies might have influenced these results, as there are no randomized controlled trials yet investigating this subject. Also, the studies used various beta blockers with different types of subjects in different tumour settings, leading to significant heterogeneity in the results.

### Other drugs

Some other cardioactive drugs are currently under investigation: while treatment with diuretics does not seem to affect tumour incidence [141], the use of statins [142], aspirin [143], and metformin [144] seems to lead to a lower risk of death and tumour incidence.

### Ongoing studies

Several ongoing studies are investigating the potential implementation of beta-blockers in this specific setting (notably propranolol and carvedilol) [7].

Although various studies have suggested that ACEi/ARB have antiproliferative effects and improve survival of many types of cancers, there are also reports of increased cancer risk: because of contradictory findings from meta-analyses, the exact relationship between RAAS blockade and development of cancer and cancer subtypes remains uncertain and more studies (longitudinal prospective cohort; randomized clinical trials) are needed to demonstrate the effects of RAAS blockade in cancer, and in this setting, large randomized controlled trials are mandatory.

## Conclusions

**Fig. 2:**
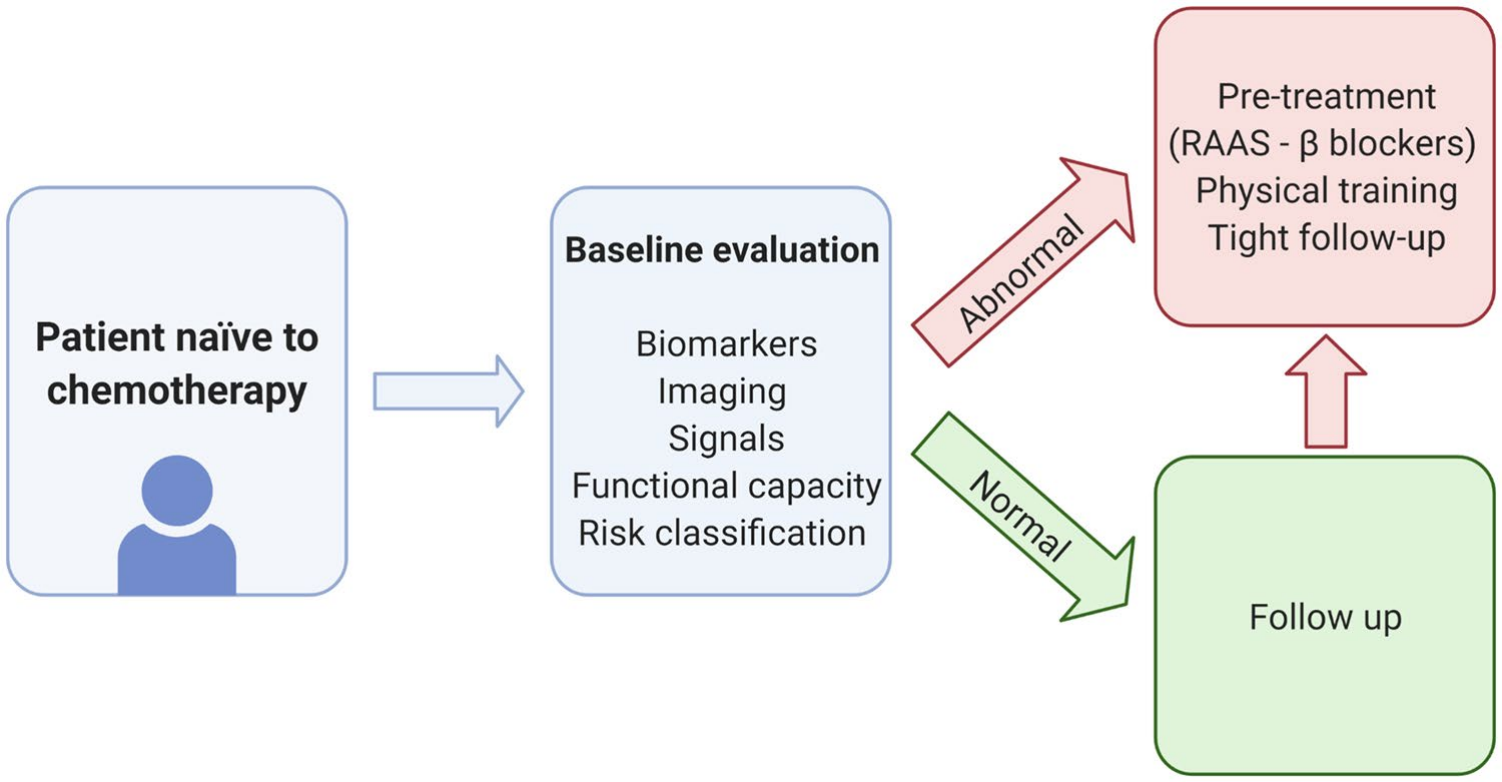
Potential cardiological clinical work-up in patients naïve to chemotherapy. Risk stratification in patients with cancer prior to chemotherapy remains pivotal. Several different methods could be employed. In the presence of preclinical alterations, a tighter cardiological work-up might be indicated

Cancer itself might be considered as a condition at risk of HF and, as such, susceptible to cardioprotective therapies even before the introduction of chemotherapy. Such primary prevention strategies would allow to avoid further complications and better risk stratify cancer patients, but, on the other hand, they imply greater risk of side effects, closer monitoring, and higher cost (Fig. 2). Overall, there are promising results from primary prevention trials investigating the cardioprotective efficacy of neurohormonal therapies [145–151]. Breast cancer patients treated with neurohormonal therapies show higher LVEF and better LV strain [121]. However, these trials have highly heterogeneous designs. The small sample size, short follow-up durations, and single-centred design highlight the need for multicentre, adequately powered randomized controlled trials. Longer follow-up duration and clinically meaningful endpoints are also required [121, 152].

Therefore, patients candidate for chemotherapy should be considered at risk of developing HF (stage A), and subclinical HF (stage B) that should be actively searched, to Initiate early an appropriate treatment and improve patient outcome.

## References

[1] Siegel RL, Miller KD, Fuchs H, Jemal A (2021) Cancer Statistics, 2021. CA Cancer J Clin 71:7–33. 10.3322/caac.21654 10.3322/caac.21654 33433946

[2] Cardinale D, Biasillo G, Cipolla CM (2016). Curing cancer saving the heart: a challenge that cardioncology should not miss. Curr Cardiol Rep.

[3] de Boer RA, Meijers WC, van der Meer P, van Veldhuisen DJ (2019). Cancer and heart disease: associations and relations. Eur J Heart Fail.

[4] Meijers WC, de Boer RA (2019). Common risk factors for heart failure and cancer. Cardiovasc Res.

[5] Koene RJ, Prizment AE, Blaes A, Konety SH (2016). Shared risk factors in cardiovascular disease and cancer. Circulation.

[6] Tu H, Wen CP, Tsai SP, Chow WH, Wen C, Ye Y (2018). Cancer risk associated with chronic diseases and disease markers: prospective cohort study. BMJ.

[7] de Boer RA, Hulot JS, Gabriele Tocchetti C, Aboumsallem JP, Ameri P, Anker SD (2020). Common mechanistic pathways in cancer and heart failure. Eur J Heart Fail.

[8] Karlstaedt A, Zhang X, Vitrac H, Harmancey R, Vasquez H, Wang JH (2016). Oncometabolite d-2-hydroxyglutarate impairs alpha-ketoglutarate dehydrogenase and contractile function in rodent heart. Proc Natl Acad Sci U S A.

[9] Cuomo A, Pirozzi F, Attanasio U, Franco R, Elia F, De Rosa E (2020). Cancer risk in the heart failure population: epidemiology mechanisms and clinical implications. Curr Oncol Rep.

[10] Strongman H, Gadd S, Matthews A, Mansfield KE, Stanway S, Lyon AR (2019). Medium and long-term risks of specific cardiovascular diseases in survivors of 20 adult cancers: a population-based cohort study using multiple linked UK electronic health records databases. Lancet.

[11] Bradshaw PT, Stevens J, Khankari N, Teitelbaum SL, Neugut AI, Gammon MD (2016). Cardiovascular disease mortality among breast cancer survivors. Epidemiology.

[12] Chow EJ, Chen Y, Hudson MM, Feijen EAM, Kremer LC, Border WL (2018). Prediction of ischemic heart disease and stroke in survivors of childhood cancer. J Clin Oncol.

[13] Cuddy SAM, Falk RH (2020). Amyloidosis as a systemic disease in context. Can J Cardiol.

[14] Zhang R, Gupta D, Albert SG (2017). Pheochromocytoma as a reversible cause of cardiomyopathy: analysis and review of the literature. Int J Cardiol.

[15] Mobine HR, Baker AB, Wang L, Wakimoto H, Jacobsen KC, Seidman CE (2009). Pheochromocytoma-induced cardiomyopathy is modulated by the synergistic effects of cell-secreted factors. Circ Heart Fail.

[16] Dero I, De Pauw M, Borbath I, Delaunoit T, Demetter P, Demolin G (2009). Carcinoid heart disease—a hidden complication of neuroendocrine tumours. Acta gastro-enterol Belg.

[17] Vitale G, Pivonello R, Lombardi G, Colao A (2004). Cardiac abnormalities in acromegaly Treat Endocrinol.

[18] Mantovani A, Allavena P, Sica A, Balkwill F (2008). Cancer-related inflammation. Nature.

[19] Zhang S, Yang X, Wang L, Zhang C (2018). Interplay between inflammatory tumor microenvironment and cancer stem cells. Oncol Lett.

[20] Gunter MJ, Stolzenberg-Solomon R, Cross AJ, Leitzmann MF, Weinstein S, Wood RJ (2006). A prospective study of serum C-reactive protein and colorectal cancer risk in men. Cancer Res.

[21] Xu J, Ye Y, Zhang H, Szmitkowski M, Makinen MJ, Li P et al (2016) Diagnostic and prognostic value of serum interleukin-6 in colorectal cancer. Medicine 95(2):e2502 10.1097/MD.0000000000002502 PMC4718291 26765465

[22] Yan G, Liu T, Yin L, Kang Z, Wang L (2018). Levels of peripheral Th17 cells and serum Th17-related cytokines in patients with colorectal cancer: a meta-analysis. Cell Mol Biol.

[23] Müerköster S, Wegehenkel K, Arlt A, Witt M, Sipos B, Kruse ML (2004). Tumor stroma interactions induce chemoresistance in pancreatic ductal carcinoma cells involving increased secretion and paracrine effects of nitric oxide and interleukin-1beta. Cancer Res.

[24] Bertero E, Canepa M, Maack C, Ameri P (2018). Linking heart failure to cancer: background evidence and research perspectives. Circulation.

[25] Ridker PM, MacFadyen JG, Thuren T, Everett BM, Libby P, Glynn RJ (2017). Effect of interleukin-1beta inhibition with canakinumab on incident lung cancer in patients with atherosclerosis: exploratory results from a randomised double-blind placebo-controlled trial. Lancet.

[26] Canli O, Nicolas AM, Gupta J, Finkelmeier F, Goncharova O, Pesic M (2017). Myeloid cell-derived reactive oxygen species induce epithelial mutagenesis. Cancer Cell.

[27] Hayes JD, Dinkova-Kostova AT, Tew KD (2020). Oxidative stress in cancer. Cancer Cell.

[28] Srinivas US, Tan BWQ, Vellayappan BA, Jeyasekharan AD (2019) ROS and the DNA damage response in cancer. Redox Biol 25:101084 10.1016/j.redox.2018.101084 PMC6859528 30612957

[29] Waris G, Ahsan H (2006). Reactive oxygen species: role in the development of cancer and various chronic conditions. J Carcinog.

[30] Maya-Mendoza A, Ostrakova J, Kosar M, Hall A, Duskova P, Mistrik M (2015). Myc and Ras oncogenes engage different energy metabolism programs and evoke distinct patterns of oxidative and DNA replication stress. Mol Oncol.

[31] Aimo A, Castiglione V, Borrelli C, Saccaro LF, Franzini M, Masi S (2020). Oxidative stress and inflammation in the evolution of heart failure: from pathophysiology to therapeutic strategies. Eur J Prev Cardiol.

[32] Tsutsui H, Kinugawa S, Matsushima S (2011). Oxidative stress and heart failure. Am J Physiol Heart Circ Physiol.

[33] Ager EI, Neo J, Christophi C (2008). The renin-angiotensin system and malignancy. Carcinogenesis.

[34] Hanif K, Bid HK, Konwar R (2010). Reinventing the ACE inhibitors: some old and new implications of ACE inhibition. Hypertens Res.

[35] Deshayes F, Nahmias C (2005). Angiotensin receptors: a new role in cancer?. Trends Endocrinol Metab.

[36] Daemen MJ, Lombardi DM, Bosman FT, Schwartz SM (1991). Angiotensin II induces smooth muscle cell proliferation in the normal and injured rat arterial wall. Circ Res.

[37] Buharalioglu CK, Song CY, Yaghini FA, Ghafoor HU, Motiwala M, Adris T (2011). Angiotensin II-induced process of angiogenesis is mediated by spleen tyrosine kinase via VEGF receptor-1 phosphorylation. Am J Physiol Heart Circ Physiol.

[38] Li SH, Lu HI, Chang AY, Huang WT, Lin WC, Lee CC (2016). Angiotensin II type I receptor (AT1R) is an independent prognosticator of esophageal squamous cell carcinoma and promotes cells proliferation via mTOR activation. Oncotarget.

[39] Afify SM, Seno M (2019). Conversion of stem cells to cancer stem cells: undercurrent of cancer initiation. Cancer.

[40] Munro MJ, Wickremesekera AC, Davis PF, Marsh R, Tan ST (2017). Renin-angiotensin system and cancer: a review. Integr Cancer Sci Therap.

[41] Unger T, Li J (2004). The role of the renin-angiotensin-aldosterone system in heart failure. J Renin Angiotensin Aldosterone Syst.

[42] Sayer G, Bhat G (2014). The renin-angiotensin-aldosterone system and heart failure. Cardiol Clin.

[43] Argyriou AA, Bruna J, Marmiroli P, Cavaletti G (2012). Chemotherapy-induced peripheral neurotoxicity (CIPN): an update. Crit Rev Oncol Hematol.

[44] Coumbe BGT, Groarke JD (2018). Cardiovascular autonomic dysfunction in patients with cancer. Curr Cardiol Rep.

[45] Teng AE, Noor B, Ajijola OA, Yang EH (2021). Chemotherapy and radiation-associated cardiac autonomic dysfunction. Curr Oncol Rep.

[46] Magnon C, Hall SJ, Lin J, Xue X, Gerber L, Freedland SJ (2013). Autonomic nerve development contributes to prostate cancer progression. Science.

[47] Hara MR, Kovacs JJ, Whalen EJ, Rajagopal S, Strachan RT, Grant W (2011). A stress response pathway regulates DNA damage through beta2-adrenoreceptors and beta-arrestin-1. Nature.

[48] Nilsson MB, Armaiz-Pena G, Takahashi R, Lin YG, Trevino J, Li Y (2007). Stress hormones regulate interleukin-6 expression by human ovarian carcinoma cells through a Src-dependent mechanism. J Biol Chem.

[49] Shahzad MM, Arevalo JM, Armaiz-Pena GN, Lu C, Stone RL, Moreno-Smith M (2010). Stress effects on FosB- and interleukin-8 (IL8)-driven ovarian cancer growth and metastasis. J Biol Chem.

[50] Bucsek MJ, Qiao G, MacDonald CR, Giridharan T, Evans L, Niedzwecki B (2017). β-adrenergic signaling in mice housed at standard temperatures suppresses an effector phenotype in CD8(+) T cells and undermines checkpoint inhibitor therapy. Cancer Res.

[51] Nissen MD, Sloan EK, Mattarollo SR (2018). Beta-adrenergic signaling impairs antitumor CD8(+) T-cell responses to B-cell lymphoma immunotherapy. Cancer Immunol Res.

[52] Sood AK, Armaiz-Pena GN, Halder J, Nick AM, Stone RL, Hu W (2010). Adrenergic modulation of focal adhesion kinase protects human ovarian cancer cells from anoikis. J Clin Invest.

[53] Li Y, Yang S, Sadaoui NC, Hu W, Dasari SK, Mangala LS et al (2020) Sustained adrenergic activation of YAP1 induces anoikis resistance in cervical cancer cells. iScience 23(7):101289 10.1016/j.isci.2020.101289 PMC7334594 32623336

[54] Thaker PH, Han LY, Kamat AA, Arevalo JM, Takahashi R, Lu C (2006). Chronic stress promotes tumor growth and angiogenesis in a mouse model of ovarian carcinoma. Nat Med.

[55] Yang EV, Sood AK, Chen M, Li Y, Eubank TD, Marsh CB (2006). Norepinephrine up-regulates the expression of vascular endothelial growth factor matrix metalloproteinase (MMP)-2 and MMP-9 in nasopharyngeal carcinoma tumor cells. Cancer Res.

[56] Cole SW, Sood AK (2012). Molecular pathways: beta-adrenergic signaling in cancer. Clin Cancer Res.

[57] Sloan EK, Priceman SJ, Cox BF, Yu S, Pimentel MA, Tangkanangnukul V (2010). The sympathetic nervous system induces a metastatic switch in primary breast cancer. Cancer Res.

[58] Partecke LI, Speerforck S, Kading A, Seubert F, Kuhn S, Lorenz E (2016). Chronic stress increases experimental pancreatic cancer growth reduces survival and can be antagonised by beta-adrenergic receptor blockade. Pancreatology.

[59] Nagaraja AS, Sadaoui NC, Lutgendorf SK, Ramondetta LM, Sood AK (2013). β-blockers: a new role in cancer chemotherapy?. Expert Opin Investig Drugs.

[60] Peixoto R, Pereira MdL, Oliveira M (2020). Beta-blockers and cancer: where are we?. Pharmaceuticals.

[61] Floras JS, Ponikowski P (2015). The sympathetic/parasympathetic imbalance in heart failure with reduced ejection fraction. Eur Heart J.

[62] Vergaro G, Aimo A, Prontera C, Ghionzoli N, Arzilli C, Zyw L (2019). Sympathetic and renin-angiotensin-aldosterone system activation in heart failure with preserved mid-range and reduced ejection fraction. Int J Cardiol.

[63] van Bilsen M, Patel HC, Bauersachs J, Böhm M, Borggrefe M, Brutsaert D (2017). The autonomic nervous system as a therapeutic target in heart failure: a scientific position statement from the Translational Research Committee of the Heart Failure Association of the European Society of Cardiology. Eur J Heart Fail.

[64] Gibson CJ, Steensma DP (2018). New insights from studies of clonal hematopoiesis. Clin Cancer Res.

[65] Acuna-Hidalgo R, Sengul H, Steehouwer M, van de Vorst M, Vermeulen SH, Kiemeney L (2017). Ultra-sensitive sequencing identifies high prevalence of clonal hematopoiesis-associated mutations throughout adult life. Am J Hum Genet.

[66] Jaiswal S, Natarajan P, Silver AJ, Gibson CJ, Bick AG, Shvartz E (2017). Clonal hematopoiesis and risk of atherosclerotic cardiovascular disease. New Engl J Med.

[67] Libby P (2017). Interleukin-1 beta as a target for atherosclerosis therapy: biological basis of CANTOS and beyond. J Am Coll Cardiol.

[68] Dorsheimer L, Assmus B, Rasper T, Ortmann CA, Ecke A, Abou-El-Ardat K (2019). Association of mutations contributing to clonal hematopoiesis with prognosis in chronic ischemic heart failure. JAMA Cardiol.

[69] Cremer S, Schloss MJ, Vinegoni C, Foy BH, Zhang S, Rohde D (2020). Diminished reactive hematopoiesis and cardiac inflammation in a mouse model of recurrent myocardial infarction. J Am Coll Cardiol.

[70] Jaiswal S, Fontanillas P, Flannick J, Manning A, Grauman PV, Mar BG (2014). Age-related clonal hematopoiesis associated with adverse outcomes. New Engl J Med.

[71] Collins RRJ, Patel K, Putnam WC, Kapur P, Rakheja D (2017). Oncometabolites: a new paradigm for oncology metabolism and the clinical laboratory. Clin Chem.

[72] Akbay EA, Moslehi J, Christensen CL, Saha S, Tchaicha JH, Ramkissoon SH (2014). D-2-hydroxyglutarate produced by mutant IDH2 causes cardiomyopathy and neurodegeneration in mice. Genes Dev.

[73] Liberti MV, Locasale JW (2016). The Warburg effect: how does it benefit cancer cells?. Trends Biochem Sci.

[74] Chen Z, Liu M, Li L, Chen L (2018). Involvement of the Warburg effect in non-tumor diseases processes. J Cell Physiol.

[75] Taegtmeyer H, Karlstaedt A, Rees ML, Davogustto G (2017). Oncometabolic tracks in the heart. Circ Res.

[76] Rees ML, Subramaniam J, Li Y, Hamilton DJ, Frazier OH, Taegtmeyer H (2015). A PKM2 signature in the failing heart. Biochem Biophys Res Commun.

[77] Sarhene M, Wang Y, Wei J, Huang Y, Li M, Li L (2019). Biomarkers in heart failure: the past, current and future. Heart Fail Rev.

[78] Curigliano G, Lenihan D, Fradley M, Ganatra S, Barac A, Blaes A (2020). Management of cardiac disease in cancer patients throughout oncological treatment: ESMO consensus recommendations. Ann Oncol.

[79] Plana JC, Galderisi M, Barac A, Ewer MS, Ky B, Scherrer-Crosbie M (2014). Expert consensus for multimodality imaging evaluation of adult patients during and after cancer therapy: a report from the American Society of Echocardiography and the European Association of Cardiovascular Imaging. Eur Heart J Cardiovasc Imaging.

[80] Zamorano JL, Lancellotti P, Rodriguez Munoz D, Aboyans V, Asteggiano R, Galderisi M (2016). 2016 ESC Position Paper on cancer treatments and cardiovascular toxicity developed under the auspices of the ESC Committee for Practice Guidelines: the task force for cancer treatments and cardiovascular toxicity of the European Society of Cardiology (ESC). Eur Heart J.

[81] Lyon AR (2015). Disparate worlds drawing closer together: cardiovascular biomarkers predict cancer outcomes in treatment-naive patients. Heart.

[82] Nikitenko LL, Fox SB, Kehoe S, Rees MC, Bicknell R (2006). Adrenomedullin and tumour angiogenesis. Br J Cancer.

[83] Nelson J, Bagnato A, Battistini B, Nisen P (2003). The endothelin axis: emerging role in cancer. Nat Rev Cancer.

[84] Law C, Glover C, Benson K, Guglin M (2010). Extremely high brain natriuretic peptide does not reflect the severity of heart failure. Congest Heart Fail.

[85] Wigle DA, Campling BG, Sarda IR, Shin SH, Watson JD, Frater Y (1995). ANP secretion from small cell lung cancer cell lines: a potential model of ANP release. Am J Physiol.

[86] Ohsaki Y, Gross AJ, Le PT, Oie H, Johnson BE (1999). Human small cell lung cancer cells produce brain natriuretic peptide. Oncology.

[87] Popat J, Rivero A, Pratap P, Guglin M (2013). What is causing extremely elevated amino terminal brain natriuretic peptide in cancer patients?. Congest Heart Fail.

[88] Petricciuolo S, Delle Donne MG, Aimo A, Chella A, De Caterina R (2021) Pre-treatment high-sensitivity troponin T for the short-term prediction of cardiac outcomes in patients on immune checkpoint inhibitors. Eur J Clin Invest 51(4):e13400 10.1111/eci.13400 32894777

[89] Florido R, Lee AK, McEvoy JW, Hoogeveen RC, Koton S, Vitolins MZ (2019). Cancer survivorship and subclinical myocardial damage. Am J Epidemiol.

[90] Missov E, Calzolari C, Davy JM, Leclercq F, Rossi M, Pau B (1997). Cardiac troponin I in patients with hematologic malignancies. Coron Artery Dis.

[91] Cramer L, Hildebrandt B, Kung T, Wichmann K, Springer J, Doehner W (2014). Cardiovascular function and predictors of exercise capacity in patients with colorectal cancer. J Am Coll Cardiol.

[92] Pavo N, Raderer M, Hulsmann M, Neuhold S, Adlbrecht C, Strunk G (2015). Cardiovascular biomarkers in patients with cancer and their association with all-cause mortality. Heart.

[93] Curigliano G, Cardinale D, Dent S, Criscitiello C, Aseyev O, Lenihan D (2016). Cardiotoxicity of anticancer treatments: epidemiology, detection, and management. CA Cancer J Clin.

[94] Zamorano JL, Lancellotti P, Rodriguez Munoz D, Aboyans V, Asteggiano R, Galderisi M (2017). 2016 ESC Position Paper on cancer treatments and cardiovascular toxicity developed under the auspices of the ESC Committee for Practice Guidelines: the task force for cancer treatments and cardiovascular toxicity of the European Society of Cardiology (ESC). Eur J Heart Fail.

[95] Mornos C, Manolis AJ, Cozma D, Kouremenos N, Zacharopoulou I, Ionac A (2014). The value of left ventricular global longitudinal strain assessed by three-dimensional strain imaging in the early detection of anthracyclinemediated cardiotoxicity. Hellenic J Cardiol.

[96] Narayan HK, Finkelman B, French B, Plappert T, Hyman D, Smith AM (2017). Detailed echocardiographic phenotyping in breast cancer patients: associations with ejection fraction decline, recovery, and heart failure symptoms over 3 years of follow-up. Circulation.

[97] Thavendiranathan P, Poulin F, Lim KD, Plana JC, Woo A, Marwick TH (2014) Use of myocardial strain imaging by echocardiography for the early detection of cardiotoxicity in patients during and after cancer chemotherapy: a systematic review. J Am Coll Cardiol 63(25 Pt A):2751–2768 10.1016/j.jacc.2014.01.073 24703918

[98] Brown J, Jenkins C, Marwick TH (2009). Use of myocardial strain to assess global left ventricular function: a comparison with cardiac magnetic resonance and 3-dimensional echocardiography. Am Heart J.

[99] Liu J, Banchs J, Mousavi N, Plana JC, Scherrer-Crosbie M, Thavendiranathan P (2018). Contemporary role of echocardiography for clinical decision making in patients during and after cancer therapy. JACC Cardiovasc Imaging.

[100] Tadic M, Genger M, Baudisch A, Kelle S, Cuspidi C, Belyavskiy E (2018). Left ventricular strain in chemotherapy-naive and radiotherapy-naive patients with cancer. Can J Cardiol.

[101] Tadic M, Baudisch A, Haßfeld S, Heinzel F, Cuspidi C, Burkhardt F (2018). Right ventricular function and mechanics in chemotherapy- and radiotherapy-naïve cancer patients. Int J Cardiovasc Imaging.

[102] Jeong D, Gladish G, Chitiboi T, Fradley MG, Gage KL, Schiebler ML (2019). MRI in cardio-oncology: a review of cardiac complications in oncologic care. J Magn Reson Imaging.

[103] Thavendiranathan P, Wintersperger BJ, Flamm SD, Marwick TH (2013). Cardiac MRI in the assessment of cardiac injury and toxicity from cancer chemotherapy: a systematic review. Circ Cardiovasc Imaging.

[104] Ferreira VM, Piechnik SK, Dall’Armellina E, Karamitsos TD, Francis JM, Choudhury RP,  (2012). Non-contrast T1-mapping detects acute myocardial edema with high diagnostic accuracy: a comparison to T2-weighted cardiovascular magnetic resonance. J Cardiovasc Magn Reson.

[105] Galán-Arriola C, Lobo M, Vílchez-Tschischke JP, López GJ, de Molina-Iracheta A, Pérez-Martínez C (2019). Serial magnetic resonance imaging to identify early stages of anthracycline-induced cardiotoxicity. J Am Coll Cardiol.

[106] Meléndez GC, Jordan JH, D’Agostino RB, Vasu S, Hamilton CA, Hundley WG (2017). Progressive 3-month increase in LV myocardial ECV after anthracycline-based chemotherapy. JACC Cardiovasc Imaging.

[107] Haslbauer JD, Lindner S, Valbuena-Lopez S, Zainal H, Zhou H, D’Angelo T (2019). CMR imaging biosignature of cardiac involvement due to cancer-related treatment by T1 and T2 mapping. Int J Cardiol.

[108] Jones LW, Liang Y, Pituskin EN, Battaglini CL, Scott JM, Hornsby WE (2011). Effect of exercise training on peak oxygen consumption in patients with cancer: a meta-analysis. Oncologist.

[109] Courneya KS, Sellar CM, Stevinson C, McNeely ML, Peddle CJ, Friedenreich CM (2009). Randomized controlled trial of the effects of aerobic exercise on physical functioning and quality of life in lymphoma patients. J Clin Oncol.

[110] Howden EJ, Bigaran A, Beaudry R, Fraser S, Selig S, Foulkes S (2019). Exercise as a diagnostic and therapeutic tool for the prevention of cardiovascular dysfunction in breast cancer patients. Eur J Prev Cardiol.

[111] Jones LW, Courneya KS, Mackey JR, Muss HB, Pituskin EN, Scott JM (2012). Cardiopulmonary function and age-related decline across the breast cancer survivorship continuum. J Clin Oncol.

[112] Persky V, Dyer AR, Leonas J, Stamler J, Berkson DM, Lindberg HA (1981). Heart rate: a risk factor for cancer?. Am J Epidemiol.

[113] Thomas F, Guize L, Bean K, Benetos A (2001). Pulse pressure and heart rate: independent risk factors for cancer?. J Clin Epidemiol.

[114] Jouven X, Escolano S, Celermajer D, Empana JP, Bingham A, Hermine O et al (2011) Heart rate and risk of cancer death in healthy men. PLoS One 6(8):e21310 10.1371/journal.pone.0021310 PMC3149594 21826196

[115] Anker MS, Frey MK, Goliasch G, Bartko PE, Prausmuller S, Gisslinger H (2020). Increased resting heart rate and prognosis in treatment-naive unselected cancer patients: results from a prospective observational study. Eur J Heart Fail.

[116] Shaffer F, Ginsberg JP (2017). An overview of heart rate variability metrics and norms. Front Public Health.

[117] Catai AM, Pastre CM, Godoy MF, Silva ED, Takahashi ACM, Vanderlei LCM (2020). Heart rate variability: are you using it properly Standardisation checklist of procedures. Braz J Phys Ther.

[118] Vanderlei LC, Pastre CM, Hoshi RA, Carvalho TD, Godoy MF (2009). Basic notions of heart rate variability and its clinical applicability. Braz J Cardiovasc Surg.

[119] Kloter E, Barrueto K, Klein SD, Scholkmann F, Wolf U (2018). Heart rate variability as a prognostic factor for cancer survival—a systematic review. Front Physiol.

[120] Hu S, Lou J, Zhang Y, Chen P (2018). Low heart rate variability relates to the progression of gastric cancer. World J Surg Oncol.

[121] Vaduganathan M, Hirji SA, Qamar A, Bajaj N, Gupta A, Zaha VG, Chandra A, Haykowsky M, Ky B, Moslehi J, Nohria A, Butler J, Pandey A (2019). Efficacy of neurohormonal therapies in preventing cardiotoxicity in patients with cancer undergoing chemotherapy. J Am Coll Cardiol CardioOnc.

[122] Schmitz KH, Courneya KS, Matthews C, Demark-Wahnefried W, Galvao DA, Pinto BM (2010). American College of Sports Medicine roundtable on exercise guidelines for cancer survivors. Med Sci Sports Exerc.

[123] Scott JM, Nilsen TS, Gupta D, Jones LW (2018). Exercise therapy and cardiovascular toxicity in cancer. Circulation.

[124] Kirkham AA, Shave RE, Bland KA, Bovard JM, Eves ND, Gelmon KA (2017). Protective effects of acute exercise prior to doxorubicin on cardiac function of breast cancer patients: a proof-of-concept RCT. Int J Cardiol.

[125] Powe DG, Voss MJ, Zänker KS, Habashy HO, Green AR, Ellis IO (2010). Beta-blocker drug therapy reduces secondary cancer formation in breast cancer and improves cancer specific survival. Oncotarget.

[126] De Giorgi V, Grazzini M, Gandini S, Benemei S, Lotti T, Marchionni N (2011). Treatment with β-blockers and reduced disease progression in patients with thick melanoma. Arch Intern Med.

[127] Grytli HH, Fagerland MW, Fosså SD, Taskén KA, Håheim LL (2013). Use of β-blockers is associated with prostate cancer-specific survival in prostate cancer patients on androgen deprivation therapy. Prostate.

[128] Montoya A, Varela-Ramirez A, Dickerson E, Pasquier E, Torabi A, Aguilera R (2019). The beta adrenergic receptor antagonist propranolol alters mitogenic and apoptotic signaling in late stage breast cancer. Biomed J.

[129] Ji Y, Chen S, Xiao X, Zheng S, Li K (2012). β-blockers: a novel class of antitumor agents. Onco Targets Ther.

[130] Bustamante P, Miyamoto D, Goyeneche A, de Alba Graue PG, Jin E, Tsering T (2019). Beta-blockers exert potent anti-tumor effects in cutaneous and uveal melanoma. Cancer Med.

[131] Choi CH, Song T, Kim TH, Choi JK, Park JY, Yoon A (2014). Meta-analysis of the effects of beta blocker on survival time in cancer patients. J Cancer Res Clin Oncol.

[132] Huang CC, Chan WL, Chen YC, Chen TJ, Lin SJ, Chen JW (2011). Angiotensin II receptor blockers and risk of cancer in patients with systemic hypertension. Am J Cardiol.

[133] Rao GA, Mann JR, Shoaibi A, Pai SG, Bottai M, Sutton SS (2013). Angiotensin receptor blockers: are they related to lung cancer?. J Hypertens.

[134] Wang KL, Liu CJ, Chao TF, Huang CM, Wu CH, Chen TJ (2013). Long-term use of angiotensin II receptor blockers and risk of cancer: a population-based cohort analysis. Int J Cardiol.

[135] McKay RR, Rodriguez GE, Lin X, Kaymakcalan MD, Hamnvik OP, Sabbisetti VS (2015). Angiotensin system inhibitors and survival outcomes in patients with metastatic renal cell carcinoma. Clin Cancer Res.

[136] Sipahi I, Debanne SM, Rowland DY, Simon DI, Fang JC (2010). Angiotensin-receptor blockade and risk of cancer: meta-analysis of randomised controlled trials. Lancet Oncol.

[137] Bangalore S, Kumar S, Kjeldsen SE, Makani H, Grossman E, Wetterslev J (2011). Antihypertensive drugs and risk of cancer: network meta-analyses and trial sequential analyses of 324168 participants from randomised trials. Lancet Oncol.

[138] Sipahi I, Chou J, Mishra P, Debanne SM, Simon DI, Fang JC (2011). Meta-analysis of randomized controlled trials on effect of angiotensin-converting enzyme inhibitors on cancer risk. Am J Cardiol.

[139] Ganz PA, Habel LA, Weltzien EK, Caan BJ, Cole SW (2011). Examining the influence of beta blockers and ACE inhibitors on the risk for breast cancer recurrence: results from the LACE cohort. Breast Cancer Res Treat.

[140] Sorensen GV, Ganz PA, Cole SW, Pedersen LA, Sorensen HT, Cronin-Fenton DP (2013). Use of beta-blockers angiotensin-converting enzyme inhibitors angiotensin II receptor blockers and risk of breast cancer recurrence: a Danish nationwide prospective cohort study. J Clin Oncol.

[141] Coogan PF, Strom BL, Rosenberg L (2009). Diuretic use and the risk of breast cancer. J Hum Hypertens.

[142] Di Bello E, Zwergel C, Mai A, Valente S (2020). The innovative potential of statins in cancer: new targets for new therapies. Front Chem.

[143] Rothwell PM, Fowkes FG, Belch JF, Ogawa H, Warlow CP, Meade TW (2011). Effect of daily aspirin on long-term risk of death due to cancer: analysis of individual patient data from randomised trials. Lancet.

[144] Soranna D, Scotti L, Zambon A, Bosetti C, Grassi G, Catapano A (2012). Cancer risk associated with use of metformin and sulfonylurea in type 2 diabetes: a meta-analysis. Oncologist.

[145] Cardinale D, Colombo A, Sandri MT, Lamantia G, Colombo N, Civelli M (2006). Prevention of high-dose chemotherapy-induced cardiotoxicity in high-risk patients by angiotensin-converting enzyme inhibition. Circulation.

[146] Gulati G, Heck SL, Ree AH, Hoffmann P, Schulz-Menger J, Fagerland MW (2016). Prevention of cardiac dysfunction during adjuvant breast cancer therapy (PRADA): a 2 × 2 factorial randomized placebo-controlled double-blind clinical trial of candesartan and metoprolol. Eur Heart J.

[147] Pituskin E, Mackey JR, Koshman S, Jassal D, Pitz M, Haykowsky MJ (2017). Multidisciplinary Approach to Novel Therapies in Cardio-Oncology Research (MANTICORE 101-Breast): a randomized trial for the prevention of trastuzumab-associated cardiotoxicity. J Clin Oncol.

[148] Guglin M, Krischer J, Tamura R, Fink A, Bello-Matricaria L, McCaskill-Stevens W (2019). Randomized trial of lisinopril versus carvedilol to prevent trastuzumab cardiotoxicity in patients with breast cancer. J Am Coll Cardiol.

[149] Lee M, Chung WB, Lee JE, Park CS, Park WC, Song BJ (2021). Candesartan and carvedilol for primary prevention of subclinical cardiotoxicity in breast cancer patients without a cardiovascular risk treated with doxorubicin. Cancer Med.

[150] Avila MS, Ayub-Ferreira SM, de Barros Wanderley Jr MR, das Dores Cruz F, Goncalves Brandao SM, Rigaud VOC,  (2018). Carvedilol for prevention of chemotherapy-related cardiotoxicity: the CECCY trial. J Am Coll Cardiol.

[151] Boekhout AH, Gietema JA, Milojkovic Kerklaan B, van Werkhoven ED, Altena R, Honkoop A (2016). Angiotensin II-receptor inhibition with candesartan to prevent trastuzumab-related cardiotoxic effects in patients with early breast cancer: a randomized clinical trial. JAMA Oncol.

[152] Kikuchi R, Shah NP, Dent SF (2020). Strategies to prevent cardiovascular toxicity in breast cancer: is it ready for primetime?. J Clin Med.

